# Supplementary material for: Mucin-Protected Caco-2 Assay to Study Drug Permeation in the Presence of Complex Biorelevant Media
Source: Pharmaceutics. 2022 Mar 24;14(4):699. doi: 10.3390/pharmaceutics14040699 (PMC9032137; doi:10.3390/pharmaceutics14040699)
Supplement: Supplementary file 1 [file pharmaceutics-14-00699-s001.zip › pharmaceutics-1624568-supplementary.pdf]

pharmaceutics

Supplementary Materials: Mucin-Protected Caco-2 Assay to Study Drug Permeation in the Presence of Complex Biorelevant Media

Dong Ye, Álvaro López Mármol, Verena Lenz, Patricia Muschong, Anita Wilhelm-Alkubaisi, Manuel Weinheimer, Mirko Koziolek, Kerstin A. Sauer, Loic Laplanche and Mario Mezler

Table S1. The transition methods for each of the drugs and its internal standard (IS) in LC-MS/MS.

Analyte	Precurser ion (m/z)	Product ion (m/z)	Entrance Potential (eV)	Declustering Potential (eV)	Collision energy (eV)
ABT-199	868.2	636.1	7	130	36
Indomethacin	358.1	139.1	10	50	30
Metoprolol	268.3	116	10	80	27
Quinidine	325.3	172.1	10	90	51
Propranolol	260.2	183	10	75	27
Carvedilol	407.3	224.1	10	83	33
Indinavir	614.6	421.1	11	49	47
Furosemide	328.9	285	−9	−60	−23
Ranitidine	315.1	176	10	40	25
Sulfasalazine	399.1	316.9	10	75	30
Doxorubicin	544.3	397	12	91	17
Piroxicam	332.1	95	10	40	51
Theophylline	179.1	122	−10	−55	−30
Caffeine	195.2	138.1	10	9	76
Atenolol	267.3	190.1	10	85	28
Naproxen	231.1	185.1	10	60	20
A-12227 (IS)	272.2	156	10	65	24
